# Understanding Drug Delivery to the Brain Using Liposome-Based Strategies: Studies that Provide Mechanistic Insights Are Essential

**DOI:** 10.1208/s12248-021-00648-z

**Published:** 2021-10-28

**Authors:** Firda Juhairiyah, Elizabeth C. M. de Lange

**Affiliations:** Research Division of Systems Biomedicine and Pharmacology, Leiden Academic Centre for Drug Research, Leiden University, Einsteinweg 55, 2333 CC Leiden, The Netherlands

**Keywords:** blood-brain barrier, brain, liposomes, mechanism-based, quantitative, pharmacokinetics, unbound drug

## Abstract

Brain drug delivery may be restricted by the blood-brain barrier (BBB), and enhancement by liposome-based drug delivery strategies has been investigated. As access to the human brain is limited, many studies have been performed in experimental animals. Whereas providing interesting data, such studies have room for improvement to provide mechanistic insight into the rate and extent of specifically BBB transport and intrabrain distribution processes that all together govern CNS target delivery of the free drug. This review shortly summarizes BBB transport and current liposome-based strategies to overcome BBB transport restrictions, with the emphasis on how to determine the individual mechanisms that all together determine the time course of *free* drug brain concentrations, following their administration as such, and in liposomes. Animal studies using microdialysis providing time course information on unbound drug in plasma and brain are highlighted, as these provide the mechanistic information needed to understand BBB drug transport of the drug, and the impact of a liposomal formulations of that drug on BBB transport. Overall, these studies show that brain distribution of a drug administered as liposomal formulation depends on both drug properties and liposomal formulation characteristics. In general, evidence suggests that active transporters at the BBB, either being influx or efflux transporters, are circumvented by liposomes. It is concluded that liposomal formulations may provide interesting changes in BBB transport. More mechanistic studies are needed to understand relevant mechanisms in liposomal drug delivery to the brain, providing an improved basis for its prediction in human using animal data.

## Introduction

The BBB is formed by cerebral endothelial cells that form the barrier between blood and brain. It plays a significant role in regulating the brain microenvironment (1, 2), and restricts the distribution of many drugs to the brain (3, 4). Therefore, BBB transport plays an important role in the central nervous system (CNS) disease treatment, such as Alzheimer’s disease, Parkinson’s disease, glioma, and stroke (5). This indicates that the development of drug delivery approaches that overcome BBB restrictions to achieve drug efficacy is in high demand.

**Fig. 1:**
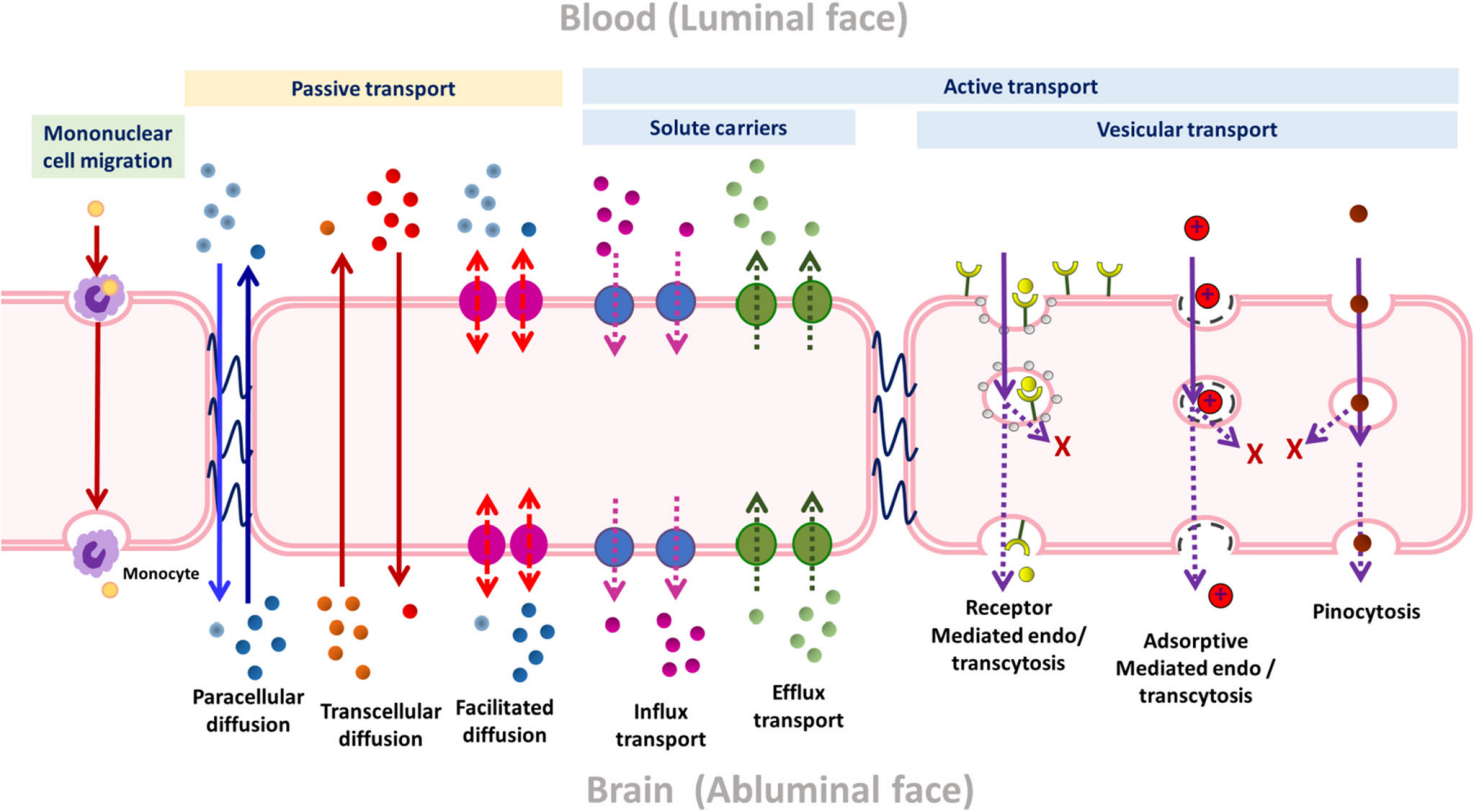
Schematic representation of transport routes across the BBB

Drug transport across the BBB is often restricted, and depends on drug properties and BBB characteristics, based in the brain cerebral endothelial cells. BBB characteristics are influenced by blood composition and contact or released factors from surrounding brains cells (pericytes, astrocytes, microglia, and neurons) (1, 2, 6). Paracellular transport is restricted by tight junctions (TJs) (7–9). Transcellular BBB transport can occur through passive diffusion as well as by active transport via influx, efflux, and vesicle-based transport modes. Vesicular transcytosis can be mediated by non-specific and specific transcytosis mechanisms, namely adsorptive-mediated transcytosis (AMT) and receptor-mediated transcytosis (RMT), respectively (2, 7, 10). An overview of BBB transport modes is provided in Fig. 1**.**

A number of approaches have been developed to overcome the problem of restricted BBB transport. One is making use of adsorptive-mediated transcytosis (AMT), receptor-mediated transcytosis (RMT), and carrier-mediated transports (CMT) (10–13) to characterize BBB transport routes. Another approach that has been investigated to increase drug delivery to the brain is nanotechnology, using nanocarriers, liposomes, vesicles, and micelles (14)(15). Liposomes are most often composed of phospholipids, especially phosphatidylcholine, but may also include other lipids, such as egg phosphatidylethanolamine. They can encapsulate both hydrophilic and hydrophobic compounds, in their biodegradable and nontoxic components (16). This liposomal vesicle, carrying the compound inside, may protect the compound from systemic degradation (17–19). Liposomes have been used in clinical practice for years for CNS disease treatment. Depocyt® is an example of an approved liposome-based formulation for the treatment of lymphomatous meningitis (20). Other examples of liposome-based formulations are Doxil® (Caelyx®) for glioblastoma multiforme and DaunoXome® for treatment of pediatric brain tumors (20–23).

The development of CNS drugs is challenging due to limited BBB transport into the brain, but also by a lack of proper distinction between BBB transport and intrabrain distribution processes, and their time dependencies and interrelationships, that altogether determine CNS target site delivery and effects (6, 24–28). Because CNS sampling in humans is ethically highly restricted, typically such mechanistic insights can only be obtained from measuring free compounds in animals, at multiple levels and with time resolution such that the rate and extent of pharmacokinetic processes can be derived. This forms the basis for proper translation from animal to human (29).

So, when using liposomal formulations, we also need to distinguish between the rate and extent of BBB transport as well as intrabrain distribution, and need appropriate animal studies to understand the impact of the liposome formulation on these processes, and how to be changed for better CNS target delivery for a particular compound.

This review first provides information on liposome-based delivery systems, and liposome types and their surface properties. This is followed by what parameters are needed to understand and to predict the rate and extent of BBB transport and intrabrain distribution, with microdialysis being the technique to obtain such parameters. This forms the basis for rationalizing liposome-based drug delivery to the brain from animal studies to be translated to the human situation.

### Liposome-Based Systems to Enhance Brain Drug Delivery

As indicated, the BBB may restrict drug distribution into the brain and liposomal formulation is expected to overcome such restrictions. Liposomes are composed of phospholipids and cholesterol formed into small spherical-shaped vesicles consisting of one or more phospholipid bilayers. In general, the components of the liposomes make them biologically inert, non-immunogenic, and biodegradable, with low inherent toxicity (16). Liposomes can be used as a carrier for biologically active compounds and have been widely used as a drug delivery system (DDS) for improving drug efficacy and eliminating drug-related toxicity or unwanted effects (17–19).

Even though liposomes have lipophilic characteristics, they are very large and cannot simply diffuse across cell membranes or between BBB cells (3). Instead, liposomes cross the BBB via transport systems, such as AMT, RMT, and CMTs (30, 31). Accordingly, liposome-based strategies can be classified as a physiological approach, in the sense that the liposome adds physiological interactions to that of the drug on its own, whereby it influences drug distribution characteristics.

**Fig. 2:**
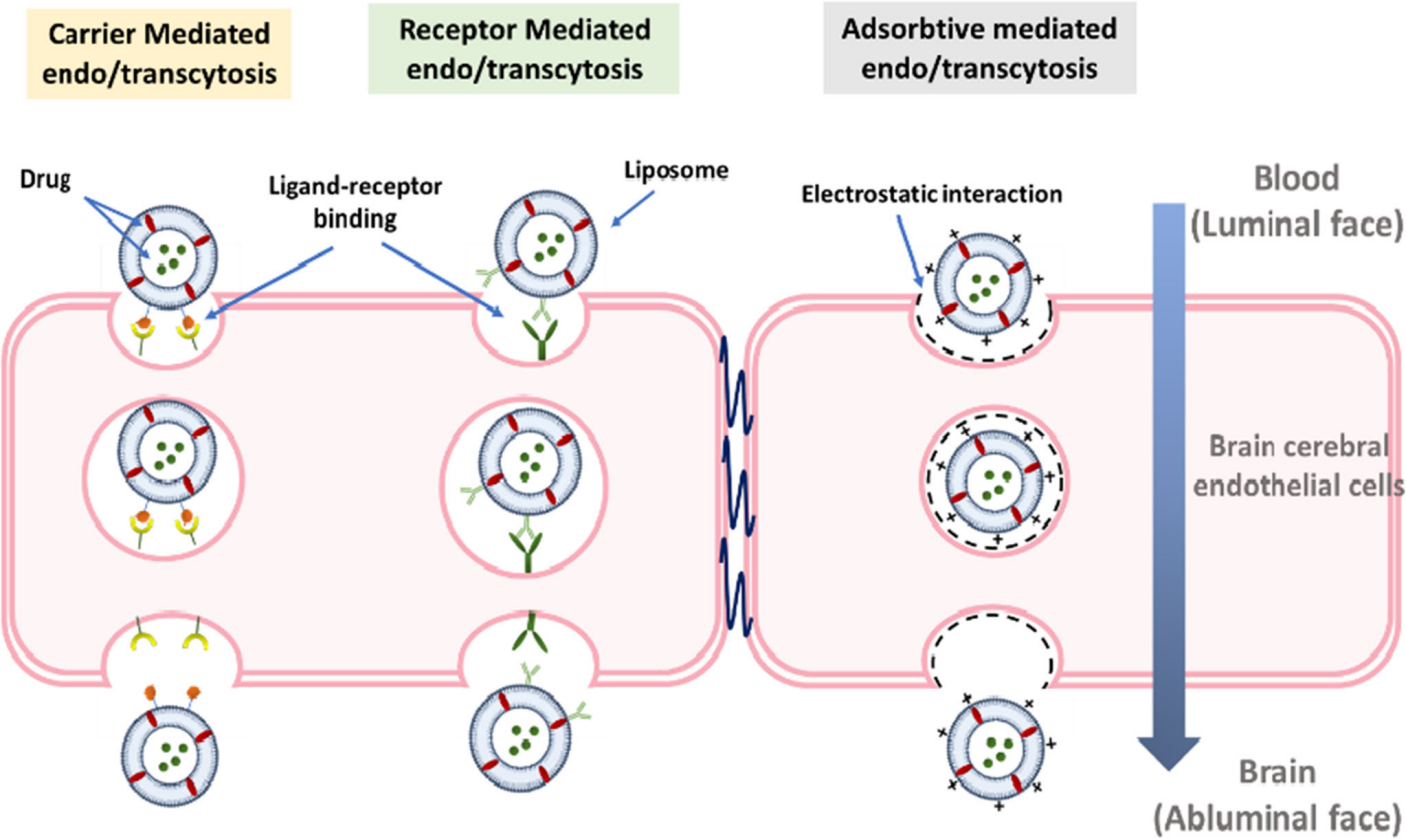
Transport routes for liposomes across the BBB and across cellular membranes. [A] Transport mechanisms of liposome across the BBB. Targeting ligand such as endogenous molecules (e.g., glucose, vitamin c, glutathione) can mediate liposome transport across BBB via CMT pathway. Meanwhile, surface modification of liposome with antibodies (e.g., OX26) mediates liposome transport across BBB via RMT pathway. Conjugation of liposome with cell-penetrating ligands or surface-charged modification can initiate liposome transport across BBB via AMT pathway. Adapted from (30)

In summary, the currently known transport modes of liposomes across the BBB are adsorptive-mediated transcytosis (AMT), receptor-mediated transcytosis (RMT), and carrier-mediated transcytosis (CMT), as shown in Fig. 2**.**

**Fig. 3:**
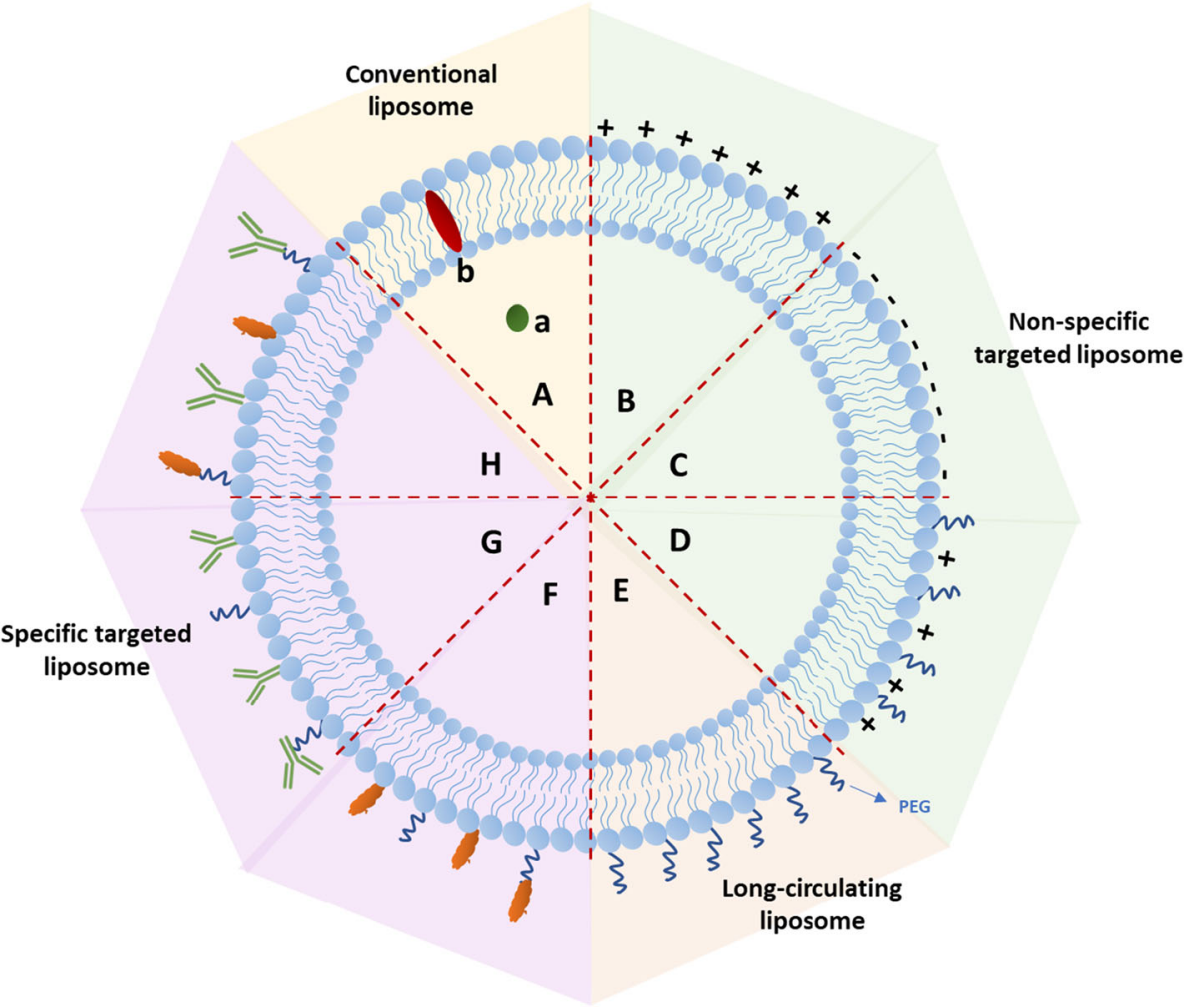
An overall development of liposome-based strategies for brain drug delivery. (Further explanation about each strategy is in Table I). [A] conventional liposome, [B] cationic liposome, [C] anionic liposome, [D] cationic PEGylated liposome, [E] long-circulating liposome, [F] targeted liposome with single functional ligand such as cell-penetrating peptides or endogenous liposome), [G] targeted liposome with single functional ligand using antibodies, and [H] targeted liposome with multiple functional ligands

In order to efficiently cross the BBB via the above-mentioned routes, instead of using conventional liposomes, further surface functionalization is possible. Here, the most recent and widely developed liposomal-based strategies utilizing BBB transport systems, e.g., cationic liposome, long-circulating liposome, and specific targeted liposome, are discussed. A general overview is given in Fig. 3 and Table I.

**Table I:** Brain Drug Delivery Improvement Using Liposome-Based Strategies

**Liposome type**	**Short description and added values**	**Considerations**	**Ref**
**Conventional liposome [A]**	▪ **Entrap hydrophilic** compound (**a**) (e.g., small molecule or biological-based compound) in the liposome’s core and lipophilic compound (**b**) in the phospholipid bilayer membrane ▪ **Stabilize compounds thus avoiding early degradation in the systemic circulation**	▪ Particle size; ▪ Compound entrapment efficiency; ▪ Liposome formulation for optimum stability and to avoid early degradation; ▪ Additional strategies are needed for optimal brain uptake.	(16, 17, 19)
**Non-specific targeted liposome**	▪ **Cationic liposome [B]** can increase drug transport across the BBB by maximizing liposome-endothelial tissue retention ▪ **Lipid surface can** facilitate adsorption of polyanions, such as DNA and RNA ▪ Monocyte can bind to **anionic liposome [C]** and facilitates drug transport across the BBB via mononuclear cell migration pathway ▪ **A cationic PEGylated liposome [D]** can enhance the brain uptake by increasing plasma concentration and tissue retention.	▪ Cationic charge; ▪ Liposome size corresponds to the adhesive force of liposome to the membrane, and hemodynamic stress resulted from the blood flow; ▪ Non-specific tissue binding.	(32–38)
**Long-circulating liposome [E]**	▪ Formulated by a PEGylation process. Polyethylene glycol (PEG) acts as a shield to protect liposomes from plasma protein binding or RES uptake. Thus, it increases plasma concentration. However, PEGylation is only able to prolong liposome circulation without improving BBB penetration.	▪ PEG density; ▪ The adverse effect related to PEG, such as hand-foot syndrome (HFS); ▪ Additional surface modification with non-specificand/or specific targeting strategy is needed to cross the BBB.	(33, 39–43)
**Specific targeted liposome**	▪ Can be achieved by conjugating liposome (or PEGylated liposome) to single functional ligand (**F** and **G**) or multiple ligands (**H**) to facilitate a specific binding to the BBB surface receptors or carrier proteins; ▪ Targeted delivery leverages the delivery efficiency of liposomes to the brain; ▪ Targeted delivery improves the therapeutic index by increasing target site drug accumulation whereas decreasing peripheral toxicity. Hence, it opens a possibility for reducing dose or dosing frequency; ▪ Targeted ligands can be antibodies (**G**), cell-penetrating peptides (**F**), or endogenous molecules (**F**) (e.g., transferrin, GSH, ApoE, lactoferrin).	▪ Ligand’s density; ▪ Ligand’s affinity to the specific target; ▪ PEG density optimization for balancing shielding property and functional ligand property.	(44–53)

### Cationic Liposomes

Liposomes with a positive surface charge can mediate electrostatic interaction with negatively charged glycocalyx at the luminal BBB membrane, thus initiating AMT. There have been numerous cationic liposome-related studies improving brain tumor treatment based on this mechanism (13). Moreover, a positively charged lipid surface can facilitate adsorption of polyanions, such as DNA and RNA, and is now widely recognized for cancer treatment (32, 33).

Intraarterial (IA) administration is a prominent route for drug delivery targeting brain tumors (54). Experimental studies by Joshi *et.al.*(55, 56) showed that reducing cerebral blood flow and IA injection effectively delivered cationic liposomes into the brain and provided 4-h retention of liposomes in the brain following injection. Furthermore, it was found that the cationic liposome formulation significantly affects regional brain deposition, which resulted in an approximately 3–15-fold enhancement compared with the anionic and neutral liposomes in both healthy and diseased rats. Interestingly, brain uptake of cationic liposomes remained superior even without any additional BBB disruption, such as using focused ultrasound (34).

The molar fraction of cationic lipids and the particle size are essential for successful drug loading and delivery to the brain. Joshi and colleagues (35) showed that the maximum cationic charge was not necessary to facilitate optimal brain tumor uptake; a modest cationic lipid molar fraction was optimal. It should be noted that an optimal molar fraction of cationic liposome formulation in one condition may be different from that in another condition or for another drug.

For better binding and subsequent increase in brain tissue uptake, the liposome adhesion force to the BBB needs to overcome the hydrodynamic force resulting from cerebral blood flow (36). For example, in a low wall shear rate created from transient cerebral hypoperfusion during IA injection, a larger cationic liposome (around 200 nm) was more favorable for tissue retention (37). However, with a faster blood flow where hemodynamic stress is high, a smaller liposome size may be preferred. Different administration routes and diseased conditions can alter the blood flow rate. Hence, liposome particle size optimization is required by considering the possible hemodynamic stress factor.

Compared to IA injection, intravenous (IV) injection is a more commonly used administration route for liposome-based delivery to the brain. However, the regional cationic liposome delivery efficiency of IA is superior compared to IV in the presence (34) or absence (57) of BBB permeability altered conditions. There are several notable reasons why IA is to be preferred over IV injection: (i) IA delivery minimizes plasma protein binding and the time available for clearance of liposomes and therefore maximizes the interaction with the exposed tissue surface, (ii) IA delivery reduces non-specific interactions of the liposome with immune cells, and (iii) IA delivery avoids non-specific uptake of cationic liposomes by the spleen and liver (39). Using IV injection, other tricks are needed to circumvent uptake of liposomes by these tissues, such as modification of the liposome surface by polyethylene glycol (PEG) ylation or targeting ligands.

### Long-Circulating Liposomes

The liposome size range influences BBB retention and thereby its delivery to the brain. Negatively and positively charged conventional liposomes have been shown to extensively interact with the cell surface, more than neutral-charged liposomes. Compared to anionic liposomes, cationic liposomes have a higher drug deposition to brain tissue (34). However, both conventional liposomes or liposomes with high charge density are susceptible to rapid clearance and reticuloendothelial system (RES) uptake from the systemic circulation (i.e., bloodstream). Other surface modifications that prolong circulation, leading to higher brain uptake, include the covalent conjugation of PEG to the liposome surface. This protects liposomes from protein plasma binding, and prevents the opsonization activity and subsequent liposomal clearance (58).

The effectiveness of long-circulating liposomes to enhance antitumor effects has been proven in several *in vitro* and *in vivo* studies (40, 59–61). A study by Hu (61) evaluated the pharmacokinetic parameters of quercetin combined with temozolomide using a tissue homogenate method. The liposomal formulation of this combination therapy prolonged drug circulation, increased the plasma concentration, and improved biodistribution into the brain compared to other tissues (e.g., spleen, lung, and liver).

A PEGylation of cationic liposome-plasmid DNA (pDNA) complexes (lipoplexes) successfully increased the systemic stability by preventing agglutination with erythrocytes or other proteins present in the blood. An *in vivo* model study showed that PEGylation of lipoplexes increases the drug delivery to brain tumor (33). There are more applications of PEGylated nanocarriers for brain targeting of bioactive compounds under development. A review by Gajbhiye (62) focuses on the comprehensive evaluation of this topic and provides further insight on several aspects such as biocompatibility, circulation time, and accumulation site.

PEGylated liposomes containing doxorubicin have been clinically evaluated for recurrent high-grade glioma (glioblastoma multiform, GBM) monotherapy, or in combination with temozolomide (63–66). Although giving only moderate treatment effect on GBM, it may be a way to long-term stabilization of GBM patients (63–66). Some clinical retrospective evaluations supported the tolerability of PEG-doxorubicin(DOX) liposomal drugs for relapsing GBM monotherapy (21, 41, 62–64). Thus, PEG-DOX liposomes might be potential for GBM treatment. Despite this benefit, the common drawback of existing PEGylated liposomal chemotherapeutic treatment is the hand-foot syndrome (HFS). The incidence depends on the type of drug, treatment schedule, and duration. Even though it is not considered a life-threatening toxicity, it can seriously impact quality of life, especially for older people (67). An alternative to non-PEGylated liposomes (NPL) has been made to eliminate the HFS side effect whereas still offering the benefits of PEGylated liposomes. Myocet® is an NPL containing doxorubicin that is clinically approved for metastatic breast cancer with an improved therapeutic index (68), including brain breast cancer metastasis (44). Nevertheless, the efficacy and safety of Myocet® for specific brain cancer treatments still needs to be explored and evaluated.

### Targeted Liposomes Using Various Ligands

As indicated, the use of surface-charged PEGylated liposomes aids drug delivery to the brain. However, surface-charged liposomes, such as cationic liposomes, have non-specific binding mechanisms to the targeted tissue and may undergo rapid clearance (13), whereas only PEGylated liposomes prolong only the liposome half-life without improving tissue penetration (33). Conjugation of liposomes with other functional ligands, such as targeting peptide vectors, endogenous molecules, and antibodies, can increase brain delivery efficiency. Ligands facilitate specific binding to the BBB surface receptors and subsequent transport across the BBB. Ligand density and affinity to receptors influence the cellular uptake and drug transport across the BBB (45–48). The ligand affinity is essential to avoid lysosomal degradation of the liposome (42). In combination with PEG, the optimal PEG density exploration is needed to balance between its shielding properties and the functional ligand properties (43, 49). In his review, Rip (69) indicated that the top five most studied transporters are transferrin receptor, GLUT-1, LDL receptor, LRP, and GSH transporter. In addition, a review by Torchilin (30) provides the chemical reactions for ligand conjugation to the liposome surface that can be used as further reference for the coupling strategy.

#### Transferrin Receptor

Liposomes targeted to transferrin receptor (TfR) are the most investigated, because of the abundance of TfR on the BBB (69). A recent study evaluated the improvement of bioavailability and brain targeting for Alzheimer’s disease (AD) using transferrin-conjugated PEGylated liposome (70). An *in vitro* study using human brain endothelial cells showed an increased uptake across BBB of osthole, which is a coumarin compound that strengthens hippocampal neurons and neural stem cells against Aβ oligomer–induced neurotoxicity in mice (70). Liposome formulations that result in a prolonged circulation time of the drug, with the addition of transferrin, improved BBB penetration, thus increased accumulation of osthole in the mice’s brain following intravenous injection (70). A TfR-targeted peptide, such as HAIYPRH (T7), also enhances PEGylated liposome transport across the BBB in an animal study for ischemic stroke using a novel neuroprotectant (ZL006) and brain tumor model (71, 72). The OX26, a well-known antibody against rat transferrin receptor, has been used to design brain targeting immunoliposomes for years (73, 74). Fluorescently labeled OX26-immunoliposomes loaded with oxaliplatin facilitated the interaction between immunoliposomes and the BBB, leading to a prominent accumulation in brain microvessels and thereby a higher uptake into the brain than IgG immunoliposomes and the free drug (75). Targeted immunoliposomes using an antihuman TfR monoclonal antibody (MYBE/4C1) is another approach for which an *in vitro* study revealed ~4-fold higher BBB penetration of doxorubicin-loadedMYBE/4C1 immunoliposomes compared to IgG immunoliposomes, showing a promising strategy for brain cancer treatment (50).

Liposome surface functionalization can use more than one receptor targeting ligand to facilitate multiple RMT routes. Each RMT route has affinity for only one selective ligand, and saturation of receptor binding by the ligand limits the transport. Using various functional ligands on the liposome surface offers better efficiency for drug delivery to the brain. Several studies have shown an enhanced drug delivery crossing the BBB for AD therapy using multiple ligands, such as a combination of antitransferrin mAb and ligands for targeting amyloid-beta (e.g., curcumin-lipid ligand and antiamyloid-beta peptide antibody) (51). In addition, another additional targeting ligand such as peptide derivate of apolipoprotein E (ApoE) for the LDL receptor was also investigated (46)*.* Intriguingly, the presence of several ligands on the liposome surface did not affect the targeting activity of each individual ligand. Various studies have been conducted to explore the synergy mechanism of dual-targeting PEGylated liposomes by employing transferrin ligand and cationic cell-penetrating peptides (CPPs) to increase BBB penetration. A combination with CPPs (e.g., TAT (52), R8 (76), and GGRRRRRRRRR-amide(47)) improved doxorubicin penetration across BBB for glioma therapy and suggested a synergy mediated transport through RMT and AMT.

Targeted liposome formulations for gene delivery using Tf-CPP, e.g., penetratin (Pen)(77), Kaposi fibroblast growth factor (kFGF)(78), vascular endothelial-cadherin-derived peptide [pVec], pentapeptide QLPVM (79), PFVYLI peptide, and R9F2 peptide (80), all indicate an improved cell internalization and subsequently the transfection efficiency. Hence, they provide potential strategies for enhancing gene therapy for neurological disorders.

#### GLUT-1 Transporter

Expression of the transport activity of GLUTs on the BBB, especially GLUT-1, is far higher than other nutrient transport systems since the brain is in high demand of glucose as an energy source, and GLUT-1 is considered the most efficient transport system. Mannose and glucose analogues have been synthesized as GLUT-1 targeting ligands and conjugated into liposome surfaces (81, 82). The higher the number of exposed glucose residues on the liposome’s surface, the stronger the affinity to the GLUT-1(83). Mannose and cell-penetrating peptides (CPPs) conjugated to liposomes were utilized to improve targeting delivery of brain-derived neurotrophic factor (BDNF) protein (84) and ApoE2 encoding plasmid DNA (pApoE2)(85) for the AD model. Both studies showed an enhanced protein expression without any observable sign of inflammation or toxicity in mice. As GLUT-1 also mediates transport of vitamin C derivates, dual-targeting liposome composed of glucosides and vitamin C is another option for improving drug delivery to the brain. Vitamin C is a substrate for another carrier present in the brain endothelial cell surface, called the Na^+^-dependent vitamin C transporter (SCVT2). Therefore, vitamin C conjugation enhances targeting drug delivery via two different transporters.

Comparing glucose-vitamin C derivative conjugated liposome containing paclitaxel was compared to paclitaxel in a single target liposome or unbound paclitaxel, a 7-fold increase in paclitaxel brain uptake was found (86).

Since GLUT-1 and SCVT2 are bidirectional transporters, there is a possibility of drug being transported back to the blood from the brain. Xiao (87) introduced a “lock-in” function to solve this problem using a thiamine disulfide system (TDS), as shown as an additional ligand previously by Ishikura (88). Also, TDS addition to liposomes conjugated with targeting ligands to GLUT-1 and SCVT2 showed a significant increase in drug concentration in the brain compared to the control situation without TDS. Thus, this strategy may be applicable to other compatible experiments intended to target GLUT-1 and SCVT2 transporters.

#### GSH Transporters

Glutathione (GSH) is an essential endogenous tripeptide that is responsible for intracellular metabolite detoxification. The Na + dependent GSH transporter that is present in the luminal side of brain endothelial cells can facilitate GSH transport to the brain via carrier-mediated transport (89, 90). The additional conjugation of glutathione (GSH) into the surface of the liposome has been proven to increase drug availability to the brain involving a specific endocytosis pathway (clathrin-mediated transcytosis, CMT)(91), and the uptake efficiency is positively correlated to the amount of GSH on the liposome surface (92, 93). The surface conjugation of GSH does not interfere with the drug release mechanism from the liposome (94). The benefit of this strategy has been applied in several disease models, such as brain cancer (94, 95), AD (96), and multiple sclerosis (97).

GSH-PEGylated liposomal doxorubicin (2B3–101) and GSH-PEGylated liposomal methylprednisolone (MP) (2B3–2010) are two promising formulations using targeted liposomes that are currently in clinical phase evaluation. 2B3-101 is an improved formulation of the existing product Doxil®/Caelyx®, under development for brain cancer treatment. In a preclinical study, the brain uptake of 2B3-101 increased despite the comparable plasma concentration of targeted and non-targeted formulations. Based on pharmacodynamic data, the new formulation elicited a potent inhibition of brain tumor growth. Giving 5 mg/kg dose twice a week showed a significant increase in survival time by 38.5% and 16.1% compared to saline and generic, respectively (95, 98). 2B3-201 is indicated for the treatment of relapsed acute multiple sclerosis. The preclinical evaluation showed better pharmacokinetic and pharmacodynamic results compared to non-targeted liposomes and unbound MP. As a result, it gave a possibility for dose reduction and lower administration frequency, thus minimizing the toxic effect of MP (97, 99). Based on the first-in-human study that has been conducted recently, 2B3-201 is considered clinically safe, and no serious adverse events arose (100), indicating a high chance for marketing authorization of this product in the near future.

#### Other Receptors

Nicotinic acetylcholine receptors (nAChRs) can facilitate a transcytosis mechanism for drug delivery to the brain. RVG29 (peptide containing 29 amino acids) conjugated to liposome was demonstrated to have high penetration efficiency and brain uptake in murine brain and dopaminergic cells for Parkinson’s disease treatment (101). Another study showed that by using a targeted peptide with a shorter amino acid, such as D8 peptide, it could minimize concern regarding the immunocompatibility of liposomal complexes caused by IgM absorption (102). Another pathway to increase brain uptake for AD is by targeting the lactoferrin receptor (LfR) by grafting liposome surfaces with lactoferrin ligand (38). Immunocytes, specifically monocytes, can act as carriers for encapsulated drugs whereas migrating across the BBB. It is known as the “Trojan horse” approach. Negative surface liposomes exhibit strong binding with monocytes. Thus, it is shown to be effectively transported across the BBB compared to neutral liposomes (103).

More brain-specific uptake strategies can be exploited for prominent brain glioma disease treatment. Conjugation of Angiopep-2 into liposomes exhibited better targeting delivery to brain tumors via the low-density lipoprotein receptor-related protein-1 (LRP1) pathway (104). Liposomal formulation conjugated to antibodies against vascular endothelial growth factor (VEGF) and its receptor type II (VEGFR2) has shown to be a promising approach for targeted delivery to glioma cells (105). The presence of P-gp efflux protein is known to cause drug resistance in brain glioma disease. A study showed that liposome surface modification with tetrandrine leads to downregulation of P-gp expression in the BBB. Eventually, it may successfully tackle the drug resistance issue (106).

#### Miscellaneous

Several studies have been conducted to evaluate the BBB transport mechanisms of the existing GSH-PEGylated-hydrogenated soybean phosphatidylcholine (HSPC) liposome strategy (known as the G-technology®). An *in vitro* study in three different cell types, i.e., brain endothelial cells, human umbilical vein endothelial cells, and human kidney epithelial cells, using flow cytometry has confirmed the brain-specific uptake of ribavirin loaded into GSH-PEG liposomes compared to non-targeted liposomes by brain endothelial cells (91). The enhanced uptake was driven by the GSH-PEG chain through endocytosis. The uptake enhancement by GSH was then supported by microdialysis studies (discussed later in this review) which also showed that the uptake efficiency was mediated by GSH and linearly correlated to the ligand concentration presented on the liposome surface (49, 91). It is important to note that thorough evaluations of protein-membrane recognition of the GSH-PEG are pending to further understand the mechanism of the BBB transport processes.

### Understanding and Predicting BBB Transport and Intrabrain Distribution

Drug distribution into and within the brain is governed by many processes, including plasma PK, plasma protein binding, passive and active transport across the BBB (7) and once within the brain, brain extracellular fluid (ECF) bulk flow, diffusion, passive and active extracellular-intracellular exchange, and CSF turnover play a role. It is of great importance to understand the mechanisms involved in uptake into and efflux from the brain, on one hand being governed by BBB functionality in terms of passive (paracellular and transcellular) diffusion, facilitated diffusion, active influx, active efflux, and absorptive or receptor-mediated endocytosis, and, on the other hand, the influence of drug physicochemical properties and structure, and biological properties (for example being a substrate for particular transporters and enzymes). As only the free drug is able to pass through the membranes, it is the free concentration difference between brain and plasma that drives BBB transport. Likewise, it is the free concentration difference between brain ECF and the cellular cytosol that drives extra-intracellular transport. Also, for drug-target interaction, the free concentration is the driving factor (28).

The steady-state extent of transport across the BBB is driven by the relative capacity of passive transport, active uptake, and active efflux at the BBB. The frequently used Kp values refer to total brain and plasma concentration ratios that do not distinguish between the free and bound drug, whereas the free drug is available for transport across membranes and binding to targets. Several reviews have provided a comprehensive discussion on the importance of the *free* drug concentration measurement instead of total concentration (24)(6, 26). So, mechanistic information on membrane transport can only be obtained on the basis of unbound drug concentrations.

Important improvements have been made in the understanding of drug distribution into and within the brain by measuring free drug concentrations. A relatively rapid and easy assessment of free concentrations in brain tissue (brain homogenate dialysis equilibration and brain slice method (107) can be combined in the combinatory mapping approach (108) to provide brain over plasma ratio of *free* concentrations (Kpuu,BBB), and extra-intracellular unbound concentration ratios (Kpuu,cell). With the combinatory mapping approach, the relationship between plasma PK and brain PK can be obtained in a more high-throughput mode, which makes it very useful for drug discovery (109). The Kpuu,BBB can also be calculated as the ratio of the AUC(0-∞) values for free drug in the brain over that in plasma, or as the ratio of free drug BBB efflux clearance (CLout) over BBB influx clearance (CLin). Likewise, intrabrain distribution Kpuu values are needed for proper understanding of brain cell membrane and subcellular membrane transport processes (27, 110). As indicated, these approaches are based on (assumed)steady-state conditions.

*In vivo* brain microdialysis, in conjunction with serial blood sampling or blood microdialysis, can be considered as a key technique to provide time-dependent information regarding free drug concentrations. With microdialysis, both the rate and extent of drug transport and distribution processes can be determined (111–113, 125). Thus, it can be used to obtain Kpuu,BBB in conjunction with the rate of transport processes (Clin, Clout). Moreover, this can be done at multiple locations, and this feature has shown that even for a drug like acetaminophen that is not subjected to any active transport, substantial differences in pharmacokinetic profiles exist in different brain compartments. Whereas there is some limit to use this water-based technique for the highly lipophilic drugs, lots of microdialysis experiments have contributed to a boost in the understanding of drug exchange across the BBB (114–116). Especially, the use of microdialysis at multiple brain locations has provided insight into the relative contribution of CNS distribution and elimination processes to the local (differences in) CNS pharmacokinetics of a compound (117).

A comprehensive CNS drug distribution model has been developed based on multi-CNS location time course data obtained with microdialysis from animals, for nine compounds with highly different physicochemical properties. Now, good prediction of CNS drug distribution can be made on the basis of CNS physiological and drug properties (thus without the need for animal data). In this CNS physiology-based pharmacokinetic (PBPK) model, the explicit separation between drug and CNS properties makes that it can convert from one CNS to another (e.g., from animal to human), and between drugs, and therefore it is a great translational tool (118).

For assessing and understanding changes in BBB transport of drugs using liposome-based formulations, in the first instance especially such mechanism-basedmicrodialysis–based experiments are essential.

### The Need for a Mechanism-Based Approaches to Study and Rationalize Liposome-Based Drug Delivery to the Brain

**Fig. 4:**
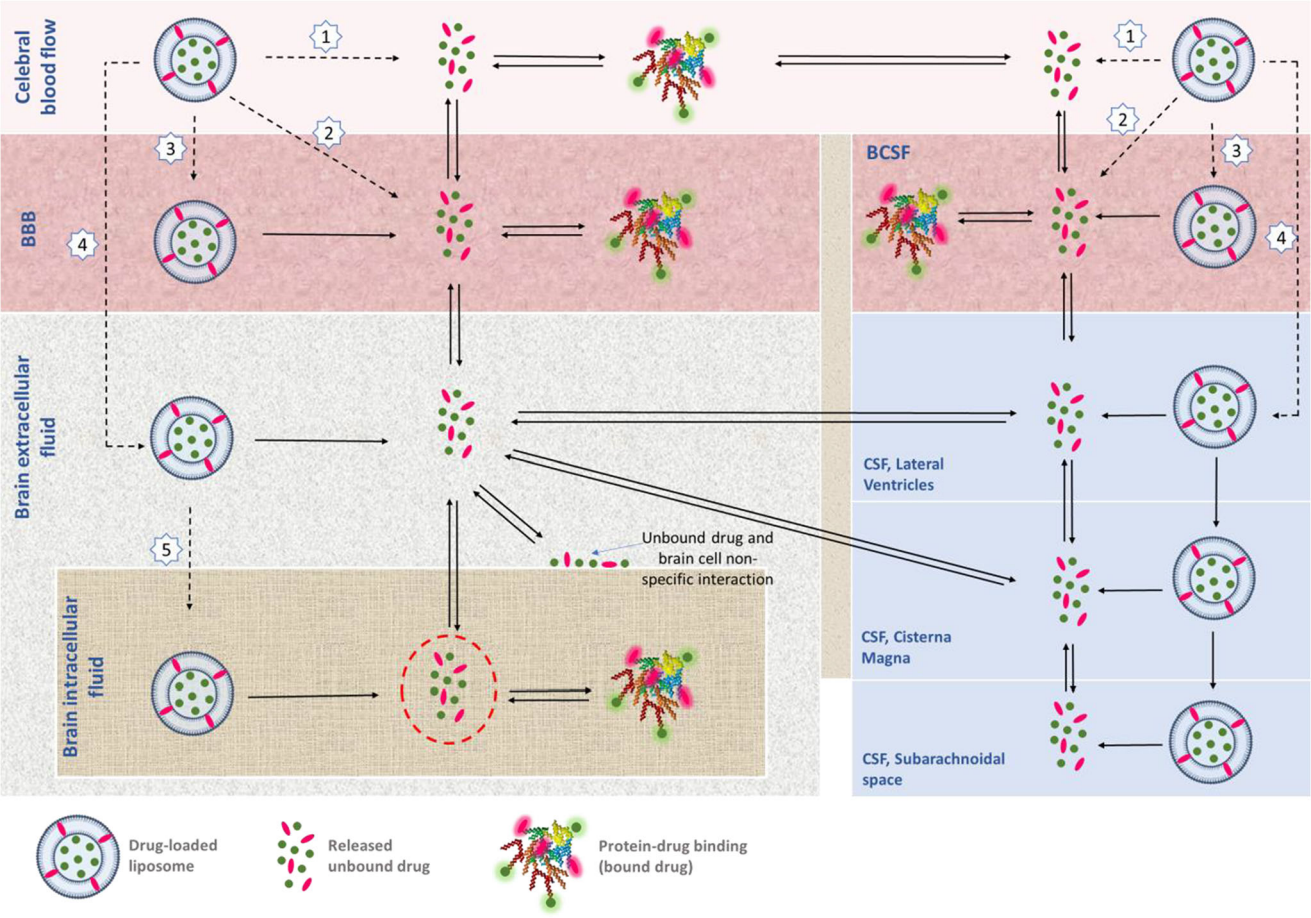
The schematic process involved in the fate of the drug using liposomal formulation for targeted brain drug delivery. For liposomal formulation, the liposomal release of the drug in plasma, liposomal transport across the BBB, and liposomal release of the drug into brain ECF should be considered on top of the plasma PK, BBB transport, and brain extracellular fluid (ECF) PK of the unbound drug. The drug can reach target site via liposome as a carrier (black dashed line) or as a released unbound drug (black full line). After intravenous administration of the liposome containing the drug, the following can happen: [1] release of the drug from the liposome in the blood and reversibly binding to plasma proteins. It is only the unbound drug that can cross the BBB or BCSFB to reach the brain ECF: [2] the liposome may fuse with BBB cell membrane and release the drug into the BBB endothelial/BCSFB epithelial cells; [3] the liposome may undergo endocytosis in BBB/BCSFB and then release the drug in endothelial cells; [4] the liposome may cross the BBB/BCSFB via transcytosis and reach the brain ECF followed by drug release, and/or likewise cross the BCSFB and reach the CSF. The released unbound drug can exchange between brain ECF, and CSF, and exchange between brain ECF and brain intracellular fluid ICF; [5] the liposome may enter the brain (ICF) then release the drug directly to the brain ICF. Only the unbound drugs that reach the ICF an available for target site binding can induce pharmacological effect (red dashed circle) Adapted from (106)

An overview of the possible processes involved in liposomal approaches for brain drug delivery is shown in Fig. 4. After intravenous administration of the liposomal formulation, the liposomes can be distributed to tissues, including the brain compartments, and can be eliminated from plasma. Then, the drug can be released from the liposomes in plasma, released from the liposomes that have gotten into the BBB cells, and released from the liposomes that have reached the brain ECF, as well as being released from the liposomes that have reached the intracellular space. Then, also the released drug itself undergoes its pharmacokinetic processes, with plasma protein binding, BBB transport and intrabrain distribution, and brain cell binding. This means that for understanding a drug target site exposure, all these mechanisms should be considered individually and then be integrated.

From the “Liposome-Based Systems to Enhance Brain Drug Delivery” section above, it is clear that there have been numerous successful proof-of-concept studies involving liposome-based drug delivery across the BBB. Relevant parameters such as brain uptake, *in vivo* drug release from liposomes, brain drug distribution, and pharmacodynamics have been quantitatively measured. In these studies, tissue homogenate has been the standard technique for evaluating the success of drug delivery to the brain, and considered the gold standard to measure drug transport efficiency parameters to the brain during the drug development stage (119).

However, studies have focused primarily on the total drug concentration ratio in the brain (homogenates) to that in plasma (Kp) as a measure for brain uptake. Alternatively, brain uptake was obtained from the plasma AUC_0-t_ and the brain permeability surface area (PS)(3, 120).

In this review, we focus on conveying the importance of mechanistic approaches to understand drug delivery to the brain with liposome-based strategies. For drug delivery to the brain following liposomal formulations, here we present changes in BBB transport and resulting brain ECF concentrations as determined by microdialysis studies in rats (49, 53, 91, 93, 96, 121–123). Quantitative evaluation of different GSH-PEG liposomal formulations on BBB transport has been studied by microdialysis for three drugs; [D-Ala^2^, *N*-MePhe^4^, Gly-ol]-enkephalin (DAMGO); diphenhydramine (DPH); and methotrexate (MTX). These are discussed below, and a summary on the resulting Kpuu,BBB values is presented in Table II.

**Table II:** Mechanism-Based Approaches Used to Determine Kpuu, Brain Values After Administration of the Drug Alone, as well as After Administration of the Drug in the Presence of Liposomes, as well as After Administration of the Drug Loaded in the Liposomal Formulation

**Liposomal formulation**	**Drug**	**Kpuu, brain values**			**Drug delivery enhancement**		**Formulation**				**Administration dosage**		**Refs**
		**Free drug**	**Coadministration free drug + liposomes**	**Drug-loaded liposome**	**Free drug + liposome-free drug**	**Free drug/drug-loaded liposome**							
		**Kpuu, free drug**	**Kpuu, free drug + liposome**	**Kpuu, drug in liposome**	**Kpuu, free + lipo control/Kpuu, drug free**	**Kpuu, drug in lipo/Kpuu, free**	**EYPC**	**HSPC**	**Cholesterol**	**PEG**	**Loading dose**	**Constant infusion**	
GSH-PEG liposomal (EYPC phospholipid)	DAMGO	0.09	*NA*	0.2	*NA*	2.3	1000 mM	N/A	750 mM	18 mM mPEG2000-DSPE (1 mol%)	75 μg/min/kg free DAMGO; 1250 μg liposomal DAMGO/min/kg for 10 min	60 μg/min/kg of free DAMGO and liposomal DAMGO for 2 h	(121)
GSH-PEG liposomal (EYPC phospholipid)	DAMGO	0.05	0.05	0.1	1.0	2.0	100 mM	N/A	750 mM	18 mM mPEG2000-DSPE (1 mol%)	75 μg/min/kg free DAMGO, liposome emulsion (1250 μg DAMGO/min/kg) for 10 min	60 μg/min/kg of free DAMGO and liposomal DAMGO for 2 h	(123)
PEG liposomal (EYPC phospholipid)	DAMGO	0.05	0.05	0.08	1.0	1.6							
PEG liposomal (EYPC phospholipid)	DPH	3.00	2.3	1.50	0.8	0.5	100 mM	N/A	66 mM	8.7 mM mPEG2000-DSPE (5 mol%)	4.5 mg/kg (150 μg/min/kg) of PEG liposomal and 4.5 mg/kg free DPH for 30 min (short infusion regiment)		(122)
PEG liposomal (EYPC phospholipid) – low dose of liposome	MTX	0.10	*NA*	0.28	*NA*	2.8	100 mM	N/A	66 mM	8.7 mM mPEG2000-DSPE (5 mol%)	2.3 mg/kg (77 mg/min/kg) and free MTX of 7.2 μg/min/kg for 30 min	2.3 mg/kg (77 mg/min/kg) and free MTX of 6 μg/min/kg for 9.5 h	(53)
PEG liposomal (EYPC phospholipid) –high dose of liposome	MTX	0.10	*NA*	0.32	*NA*	3.2					15 mg/kg (500 mg/min/kg) and free MTX of 7.2 μg/min/kg for 30 min	15 mg/kg (500 mg/min/kg) and free MTX of 6 μg/min/kg for 9.5 h	
PEG liposomal (HSPC phospholipid)	MTX	0.10	*NA*	0.11	*98*	1.1	N/A	100 mM			15 mg/kg (500 mg/min/kg) and free MTX of 7.2 μg/min/kg for 30 min	15 mg/kg (500 mg/min/kg) and free MTX of 6 μg/min/kg for 9.5 h	
PEG liposomal (EYPC phospholipid)	MTX	0.10	0.09	1.50	0.9	15.0	100 mM	N/A	66 mM	1.7 mM mPEG2000-DSPE (1 mol%)	Free MTX of 7.2 μg/min/kg and liposomal formulation 15 mg/kg for 30 min	Liposomal formulation of 15 mg/kg and free MTX of 6 μg/min/kg for 9.5 h	(49)
GSH-PEG liposomal (EYPC phospholipid)	MTX	0.10	0.09	0.53	0.9	5.3							
PEG liposomal (HSPC phospholipid)	MTX	0.10	0.09	0.23	0.9	2.3	N/A	100 mM					
GSH-PEG liposomal (HSPC phospholipid)	MTX	0.10	0.09	0.82	0.9	8.2							

*DAMGO* [D-Ala2, N-MePhe4, Gly-ol]-enkephalin, *DHP* diphenhydramine, *MTX* methotrexate

#### DAMGO

Lindquist *et al.*(121) investigated DAMGO BBB transport using (GSH-PEG) liposomes, following a 10-min and a 2-h infusion. The Kpuu,BBB value for free DAMGO was 0.09 and increased to 0.21 by using the GSH liposomes. Then, in a later study (123), the difference between (PEG) ylated liposomes, with or without the specific brain targeting ligand GSH, was investigated. Somewhat surprisingly, the GSH coating on the liposomes did not result in an additional increase in DAMGO concentrations in the brain, in contrast to earlier studies on GSH coating. The authors suggested that the drug properties in the liposomes also play a role. Anyway, the limited BBB transport of free DAMGO could be doubled by the use of PEGylated liposomes without using a specific brain targeting ligand.

#### DPH

Hu *et al.*(122) investigated how PEGylated (PEG) liposomes would influence brain delivery of diphenhydramine (DPH), a drug with active influx at the BBB, in rats. BBB transport of DPH after 30-min intravenous infusion of free DPH, PEG liposomal DPH, or free DPH + empty PEG liposomes was compared by determining the free DPH concentrations in brain ECF and plasma. Free DPH is subjected to active BBB influx transport into the brain, which appeared to be a time-dependent manner (higher active transport into the brain at earlier time point). A Kpuu,BBB value of 3.0 was found at later stages of the study. The liposomal formulation of DPH significantly decreased brain uptake of DPH, with a reduction of Kpuu,BBB to 1.5. Coadministration of empty PEG liposomes with the free DHP a Kpuu value of 2.3 was found, whereas DHP was found to bind to the liposomes. This all indicates complex BBB transport behavior of DHP in the presence of liposomes, or DHP in the liposomes.

#### MTX

Hu *et al.*(53) compared two PEGylated liposomal MTX formulations. One liposomal formulation was based on hydrogenated soy phosphatidylcholine (HSPC) and the other on egg-yolk phosphatidylcholine (EYPC). Compared with the HSPC liposomal for both high- and low-dose EYPC liposomes, a 10-fold increase of MTX release from the liposome into plasma was found. Free MTX has a low Kpuu,BBB, in this study being of 0.10 ± 0.06. The HSPC liposomes did not affect the extent of BBB transport of MTX (Kpuu,BBB was 0.11). In contrast, EYPC liposomes significantly improved the extent of MTX BBB transport with a 3-fold increase of Kpuu,BBB, which was 0.28 for high-dose EYPC liposomal MTX, and 0.32 ± 0.13 for the low-dose EYPC liposomal MTX. These findings indicate that different phospholipids in liposomal formulations may have different consequences for MTX delivery to the brain. In a next study, Hu *et al.*(49) investigated the impact of conjugation of GSH to different liposomal formulations on MTX BBB transport. GSH-PEG liposomal MTX based on HSPC or EYPC and their corresponding PEG control liposomes were compared. Free MTX had a Kpuu,BBB of 0.10, PEG-HSPC liposomes did not affect the brain uptake of MTX, whereas PEG-EYPC liposomes resulted in an increase in Kpuu,BBB to 1.5. Compared to PEG control formulations, GSH-PEG-HSPC liposomes increased the Kpuu,BBB value of MTX to 0.82, whereas GSH-coating on PEG-EYPC liposomes did not result in a further enhancement in brain uptake. The coadministration of empty GSH-PEG-HSPC liposomes with free MTX did not influence the MTX brain uptake. So, these results indicate that the brain-targeting effect of GSH-PEG liposomal MTX highly depends on the liposomal formulation that is combined with GSH. Since the EYPC liposome mechanism of BBB transport is mainly via membrane fusion (124), it was suggested that the fluidic lipid composition of EYPC (53, 123, 124) can easily fuse with endothelial cells and thus increase drug delivery of MTX to the brain.

Altogether, these studies show that the impact of brain drug delivery using liposomes not only is influenced by the liposomal composition in which the drug is encapsulated, that even empty liposomes may influence (in a drug-dependent manner) the BBB transport of the drug, and also that the BBB transport mechanisms of the free drug (being actively effluxed or influxed) have an impact on what change in the extent of BBB transport (Kpuu,BBB) is brought about. This information could never be obtained by measuring total plasma and brain concentrations, as drug binding to either plasma proteins and brain tissue components and these mechanisms should be dealt with separately from BBB transport mechanisms.

## Discussion

Liposomal drug delivery approaches are a prominent strategy to overcome BBB transport restriction. In proof-of-concept studies, it was shown that liposome surface modifications can improve the circulation time in blood, the therapeutic index, and the bioavailability, as well as change the drug distribution to the brain. However, only a few liposomal products for brain disease treatment successfully reached clinical evaluation (20). This may be caused also by the lack of understanding of the essential factors contributing to the optimum CNS drug delivery during the development program. Though limited, some studies have convincingly shown that the quantification of free drug concentrations in plasma and brain with and without liposomal formulation, and the Kpuu values that can be calculated by that, is very important to understand the mechanisms of liposomal BBB transport and consequence for brain drug delivery changes. Microdialysis is the experimental approach that can provide such information, and is recommended in preclinical research to rationalize liposome-based drug delivery to the brain, as it is clear that the use of only total plasma and total brain concentrations is often not suitable to draw proper conclusions on brain drug delivery and changes by liposomal formulations.

It remains to be important to understand the rate and extent of mechanisms that altogether determine the availability of the drug to its target in the brain, and how these rates and extents depend on liposomal formulations, but also on changes in physiology (condition) of the subject (animal, human, patient), as the basis of translation between conditions, such as from animal to human. To understand the impact of the rate and extent of such mechanisms, the “mastermind research approach” (MRA) was introduced as a systematic strategy that accounts for differences in body processes between different conditions, which should be explicitly addressed to be able to translate between these conditions (25).

The microdialysis technique has also been key for the development of the CNS physiologically based PK model (125) that is able to predict drug PK in different CNS compartments, in animals as well as in humans, using (unbound) plasma PK and drug properties only (29). This model has been further defined, and the CNS PBPK model version 3.0, and can be used as an *in silico* predictor of CNS drug distribution as well as an explorer of “WHAT IF” scenarios (111). As a future perspective, this model could be extended to also include the liposomal transport routes. This should be first based on smart data produced by MRA animal studies; also in disease conditions, this model will replace further use of animals as mechanistic knowledge will be condensed in the CNS PBPK model that can be used to predict human brain drug delivery, based on drug and liposomal formulations properties.

Furthermore, the rate and extent results obtained from microdialysis studies have proven to be valuable for (predictive)PK-PD modeling. For example, Hu *et al.*(112) evaluated the influence of (targeted) liposomal formulation on the therapeutic drug index. In this modeling approach, it was shown a non-targeted liposome improves the therapeutic index compared to the non-encapsulated by reducing peripheral toxicity. Then, the targeted liposome improves the therapeutic index by lowering the peripheral toxicity and increasing the CNS effect.

## Conclusions

Taken together, the liposomal drug delivery approach is a prominent strategy that could be used to overcome BBB transport restriction. Much progress has been made in the last years in this area; however, there is too little mechanistic understanding of the roles of drug properties, liposomal formulations, and the (patho-)physiological conditions to make general conclusions on the enhancement of the delivery of a particular drug to the brain. Especially quantitative and mechanism-based approaches, including measurements of unbound drug concentration-time profiles in blood and brain by microdialysis, have provided important insights for translational approaches to the clinic. This approach may help to accelerate liposome-based drug delivery development, and more liposomal formulation products for the treatment of human brain diseases can be successfully marketed.
